# vNOTES high uterosacral ligament suspension versus laparoscopic lateral suspension in the management of vaginal vault prolapse: initial experience in a single tertiary center

**DOI:** 10.55730/1300-0144.6218

**Published:** 2026-05-03

**Authors:** Ramazan ADAN, Fatih ŞAHİN, Ulya ÜSKENT, Mehmet Gökhan ÇULHA, Arzu Bilge TEKİN

**Affiliations:** 1 Department of Obstetrics and Gynecology, Prof. Dr. Cemil Taşçıoğlu City Hospital, İstanbul, Turkiye; 2 Department of Obstetrics and Gynecology, İstinye University Medical Park Hospital, İstanbul, Turkiye; 3 Department of Urology, University of Health Sciences, Prof. Dr. Cemil Taşçıoğlu City Hospital, İstanbul, Turkiye; 4 Department of Obstetrics and Gynecology, University of Health Sciences, Şehit Prof. Dr. İlhan Varank Training and Research Hospital, İstanbul, Turkiye

**Keywords:** Laparoscopic lateral suspension, transvaginal natural orifice transluminal endoscopic surgery, pelvic organ prolapse, uterosacral ligament suspension, vaginal vault

## Abstract

**Background/aim:**

The optimal technique for vaginal vault prolapse correction has yet to be identified. This study aimed to evaluate the anatomic and functional outcomes of women who underwent vaginal natural orifice transluminal endoscopic high uterosacral ligament suspension (vNOTES HUSLS) or laparoscopic lateral suspension (LLS).

**Materials and methods:**

This retrospective study included symptomatic women with stage ≥2 vault prolapse who underwent vNOTES HUSLS or LLS between January 2019 and June 2022 at a tertiary center. The surgical efficacy, postoperative outcomes, complication rates, and recurrence frequencies of these procedures were compared. The primary outcomes of the study included anatomical success, patient-reported (subjective) success, and recurrence rates.

**Results:**

The study included 64 women, 32 of whom underwent vNOTES HUSLS and 32 of whom underwent LLS. Demographic characteristics and preoperative Pelvic Organ Prolapse Quantification (POP-Q) values were comparable (p > 0.05). The mean Patient Global Impression of Improvement (PGI-I) score did not differ significantly between the groups (1.6 ± 0.8 vs. 1.7 ± 0.7, p = 0.344). Improvements in terms of sexual function scores (p < 0.001) were observed postoperatively in both groups. No difference was observed in postoperative sexual questionnaires scores (p > 0.05). Vault suspension by vNOTES produced better results only for point Ba (most distal portion of the remaining upper anterior side of the vaginal wall) of the POP-Q score (p = 0.03). The incidence rates and types of postoperative complications and recurrences were similar between the groups.

**Conclusion:**

Both vNOTES HUSLS and LLS safely support the vault and restore the normal vaginal anatomy with similar effectiveness. Functional results were also similar and satisfactory between the groups.

## 1. Introduction

Increasing life expectancy, coupled with the high prevalence of hysterectomy and its established role as a risk factor for pelvic organ prolapse, has coincided with a rising incidence of vaginal vault prolapse [1].

Since apical support is the mainstay of vaginal cuff restoration, sacrocolpopexy is still accepted as the gold standard technique in vaginal vault prolapse (VVP) [2,3]. However, laparoscopic lateral suspension (LLS) using mesh is an efficient alternative technique for apical support [4]. Unlike laparoscopic sacrocolpopexy, lateral suspension avoids surgery near the sacral promontory, eliminating the risk of serious complications associated with that specific surgical area [5]. Additionally, sacrocolpopexy has a 4% risk of needing a more invasive laparotomy, but the LLS procedure has not shown this complication in any reported cases. It also appears to be easier to learn [6].

Vaginal approaches have been used in cuff prolapsus surgery for many years. Uterosacral ligaments are strong native tissues used in cuff surgery and apical support. However, transvaginal uterosacral ligament suspension (USLS) was found to be associated with an approximately 4.5% risk of intraoperative ureteral kinking due to the proximity of the ureter to the suture site [7].

In recent years, vaginal natural orifice transluminal endoscopic surgery (vNOTES) has offered advantages over the traditional transvaginal approach in cuff restoration [8–10]. Direct visualization of important structures such as the rectum and ureter that cannot be obtained with the traditional transvaginal approach and more precise suture placement facilitated by confirmation of the location of the ischial spine are some of its advantages [8–10].

The optimal surgical approach for the management of posthysterectomy vaginal vault prolapse remains a subject of ongoing debate [2,11,12]. This condition, characterized by the descent of the vaginal apex due to a loss of ligamentous support, is a frequent challenge in pelvic reconstructive surgery. While there are several surgical options available, there is no clear consensus on which technique offers the best outcomes in terms of effectiveness, safety, and long-term results [2,11,12]. This highlights the need for further research to identify the optimal surgical approach for this condition.

This study aimed to evaluate the anatomic and functional outcomes of women who underwent native tissue repair via VNOTES high USLS (HUSLS) or mesh repair via LLS and to compare the effectiveness and complication rates of the two techniques.

## 2. Materials and methods

### 2.1. Patients

This retrospective study included symptomatic women with stage ≥2 vault prolapse who underwent VNOTES HUSLS or LLS between January 2019 and June 2022 at a tertiary training and research hospital. Ethics committee approval was obtained before the study began (date: 04/03/2024, reference number: 71) The study was conducted in accordance with the criteria of the Declaration of Helsinki.

The exclusion criteria were as follows: women who had previously undergone surgical treatment for pelvic organ prolapse (POP); cases in which additional surgery was needed due to anterior or posterior prolapse or stress incontinence in the same session; and women who had conditions unsuitable for surgery such as pelvic inflammatory disease, a known or suspected gynecological malignancy, rectovaginal endometriosis, or obliterated rectovaginal space detected on pelvic examination.

### 2.2. Clinical assessment and follow-up

Documented data from clinical assessments of the patients for genitourinary, sexual, and bowel disorders were examined. Medical records constituted the source of data regarding preoperative clinical assessments, baseline characteristics of the patients, and intraoperative and postoperative outcomes. Additionally, parameters such as the duration of surgery, preoperative vs. postoperative hemoglobin difference, length of hospital stay in the postoperative period, hospital readmission rate, intraoperative and postoperative complications and recurrences, and need for conversion to laparoscopy or open technique were examined.

Patients received comprehensive information regarding both surgical routes. The choice of surgery was based on shared decision-making, considering both clinical indications and patient preference.

Routine postoperative follow-up visits were performed at 6 months and annually thereafter. The Pelvic Organ Prolapse Quantification (POP-Q) system was used to stage women with vault prolapse [13]. Scores reflecting stage ≥2 prolapse of at least one compartment were defined as indicating objective recurrence, while the presence of bulging symptoms indicated subjective recurrence. The effectiveness of both procedures was evaluated by comparing preoperative and postoperative POP-Q staging. Prolapse level was assessed by comparing POP-Q scores measured before surgery and at the final follow-up appointment.

The Patient Global Impression of Improvement (PGI-I) scale, a seven-point quality of life questionnaire with possible scores ranging from 1 (“very much improved”) to 7 (“very much worse”), was used to assess patients’ postoperative satisfaction as a subjective outcome [14]. Validated versions of the Pelvic Organ Prolapse/Urinary Incontinence Sexual Questionnaire (PISQ-12) and Female Sexual Function Index (FSFI) questionnaire [15,16] were administered preoperatively and at 6 months postoperatively in the clinic. The questionnaires were administered again at the patients’ last visits. Sexual pain/discomfort was measured using the pain subdomain of the FSFI, which assesses the degree of sexual discomfort or pain following vaginal penetration (17th, 18th, and 19th items of the FSFI). The total score of the FSFI pain subdomain was calculated by summing the scores of the three items and multiplying the resulting number by 0.4, yielding a score range of 0 to 6. The Clavien–Dindo classification was used to evaluate surgical complications [17].

In the event of occult urodynamic stress urinary incontinence (SUI) or mild SUI, we discussed a two-stage intervention with the patient if necessary. An increasing number of urogynecologists are adopting this approach in relation to occult SUI, treating incontinence as a second step after repairing the prolapse only if it becomes symptomatic [6].

### 2.3. Surgical technique

#### 2.3.1. Laparoscopic lateral suspension (LLS)

LLS was performed in accordance with the technique previously described by Dubuisson et al. [18] The polypropylene mesh used had a width of 2.5 cm and a length of 25 cm. Following surgical field antisepsis, the vaginal cuff was suspended with the use of forceps holding a sponge. The trocars were then inserted and the bladder was inflated to 300 mL. Once the bladder edges were identified, careful blunt dissection was applied to develop the vesicovaginal space. In the presence of adhesions, sharp dissections were necessary. The middle part of the mesh was placed flatly in the vesicovaginal space and fixed with nonabsorbable sutures. An atraumatic laparoscopic instrument was inserted through skin incisions of approximately 2–3 mm approximately 3 cm above and 4 cm lateral to the anterior superior iliac spine, followed by perforation of only the aponeurosis of the external oblique muscle and retroperitoneal advancement of the instrument through the lateral abdominal wall. Under laparoscopic visualization and avoiding vascular areas, the laparoscopic instrument moved safely through the created bilateral tension-free retroperitoneal tunnels. The lateral arms of the mesh were secured bilaterally to the aponeurosis of the external oblique muscle and behind the anterior superior iliac spine. The distal arms at skin level were then cut, achieving a symmetrical tension-free suspension. Finally, the peritoneum was closed to allow reperitonealization of the mesh surface and to reduce mesh-related complications.

#### 2.3.2. VNOTES HUSLS

A transverse apical colpotomy was performed near the hysterectomy scar site to enter the peritoneal cavity. Following entry into the peritoneal cavity, a transvaginal retractor was inserted through the vaginal vault and the vaginal access platform was established. The ureters and uterosacral ligaments were identified via laparoscopic view. After waiting for ureteral peristalsis, the ureter was released peritoneally with laparoscopic scissors to prevent kinking. Bilateral nonabsorbable sutures were placed near the intermediate portions of the uterosacral ligament at the level of the ischial spine, constituting a total of 4 stitches (Figure). Gentle traction was then applied to the sutures to verify correct placement, and the vNOTES platform was removed and the peritoneum was closed. The sutures were fixed to the ipsilateral cardinal ligament stump and the pubocervical fascia on the anterior wall. Finally, the aforementioned nonabsorbable sutures were attached to the vaginal cuff and tied. Routine postoperative cystoscopy was performed.

The same surgical team performed all surgical procedures as planned and executed by the authors (F.Ş. and R.A.).

The primary outcomes of the study were the anatomical and subjective success rates, together with the recurrence rate.

### 2.4. Statistical analysis

Data normality was assessed using the Kolmogorov–Smirnov test and skewness and kurtosis values. Normally distributed continuous variables were presented as mean ± standard deviation. Comparisons between two independent groups were analyzed using the independent t-test for normally distributed data and the Mann–Whitney U test for nonnormally distributed data. Paired t-tests were used for paired comparisons (preoperative vs. postoperative). Categorical variables were presented as frequency and percentage. Statistical significance was set at p < 0.05. All statistical analyses were performed using IBM SPSS Statistics 25.0 (IBM Corp., Armonk, NY, USA).

## 3. Results

This retrospective study included 64 women aged 40–80 years with symptomatic vaginal vault prolapse of stage ≥2. These patients were selected from a pool of individuals offered both vNOTES HUSLS and LLS procedures by two surgeons. Patients meeting the exclusion criteria (n = 27) were not enrolled. Of the eligible patients who consented to surgery, 32 underwent vNOTES HUSLS and 32 underwent LLS (1:1 ratio).

The mean age of the patients was 58.13 ± 11.09 years and the mean body mass index value was 27.7 ± 3.2 kg/m^2^. There was no statistically significant difference between the groups in terms of demographic characteristics or preoperative POP-Q scores (p > 0.05) (Table 1). The median follow-up time for both groups was 21 months (range: 14–30).

### 3.1. Objective evaluation of POP

Points Aa (midline of the anterior vaginal wall), Ba (most distal portion of the remaining upper anterior side of the vaginal wall), C (lowest edge of the cervix or the vaginal cuff), Ap (midline of posterior vaginal wall 3 cm proximal to hymen), and Bp (most distal portion of the remaining upper posterior side of the vaginal wall) were improved significantly after vNOTES HUSLS (p < 0.001). Significant improvements were also observed postoperatively in the LLS group for points Aa and Bp (p < 0.001). There was no statistically significant difference between the preoperative and postoperative values of the perineal body, genital hiatus, or total vaginal length parameters of the two groups (Table 2).

No apical or central recurrent prolapse was observed in either group. Anterior recurrence was noted in a total of 4 patients, with 2 in each group (6.25%). Anterior colporrhaphy was performed for these patients due to the occurrence of symptomatic findings at the 6th postoperative month. Intraoperative complications included bladder injury in a total of 4 patients, with 2 in each group (6.3%). Perioperative management was performed for these patients. The bladder was repaired; a Foley catheter was placed before the repair and the catheter was maintained for 1 week. No significant difference was observed between the two groups in terms of operation time (p = 0.071) or intraoperative bleeding (p = 0.891). No further intervention was required and no grade ≥II postoperative complications were observed in either group according to the Clavien–Dindo classification.

### 3.2. Subjective evaluation of POP

Mean PGI-I scores did not differ significantly between the groups (1.6 ± 0.8 vs. 1.7 ± 0.7, p = 0.344) (Table 3). Statistically significant improvements were observed postoperatively in both groups in terms of PISQ-12 and FSFI scores (p < 0.001) (Table 2). There was no statistical difference between the postoperative PISQ-12 and FSFI scores for the vNOTES and LLS groups (p > 0.05) (Table 4).

Vault suspension by vNOTES HUSLS produced better results in terms of POP-Q point Ba compared to LLS (p = 0.03). Points Aa, C, Ap, and Bp were comparable between the groups without any significant difference (p > 0.05) (Table 4).

## 4. Discussion

The findings of the present study suggest that both vNOTES HUSLS and LLS are safe and effective surgical techniques for the management of vaginal vault prolapse, yielding favorable anatomical and functional outcomes over a medium-term follow-up period. Notably, no patients required secondary interventions and no postoperative complications of Clavien–Dindo grade ≥II were observed in either group.

Sacrocolpopexy, which is considered the gold-standard technique, has several drawbacks including proximity to vascular tissues, risk of mesh erosion, difficulty of application in obese women, and a longer learning curve with the laparoscopic approach. In a systematic review, the mean operation time for laparoscopic sacrocolpopexy was reported to be 213 min and the mean intraoperative blood loss was 121 mL [19]. In the present study, the duration of surgery for both the vNOTES HUSLS group and the LLS group was shorter (85.48 ± 25.65 and 91.16 ± 32.49 min, respectively). The potential complications associated with lengthy surgical procedures reflect the importance of techniques that offer both a short operation time and a shorter learning curve. Shorter operation times can reduce the risk of infection, blood loss, and adverse effects from anesthesia. A shorter learning curve means that surgeons can become proficient in the technique more quickly, leading to improved patient outcomes and potentially wider access to the procedure. This is particularly relevant for minimally invasive procedures where technical skill and efficiency are crucial.

Suspension surgeries using native tissues have become popular in the context of vaginal approaches, especially in light of the reservations about mesh specified by the U.S. Food and Drug Administration [20]. From this perspective, USLS is a well-established surgical technique for repairing both primary prolapse and vault prolapse, although the data in the literature are relatively limited compared to other techniques. Preservation of the natural orientation of the vaginal axis is a noteworthy advantage of USLS. HUSLS can be performed vaginally, laparoscopically, and robotically. Ureteral injury was observed at a rate of about 11% when the procedure was performed vaginally [21]. In the present study, no ureteral complications were observed, as meticulous ureteral dissection and routine postoperative cystoscopy were performed. Complications due to proximity to the ureter can often be reduced with careful dissection, particularly via vNOTES. In this sense, vNOTES is likely to become an increasingly popular and routine approach for vaginal hysterectomies.

Sacrospinous ligament fixation (SSLF) has been performed for many years both to restore vault prolapsus and as part of routine vaginal hysterectomy [22,23]. However, in unilateral and particularly right-sided SSLF, the optimal vaginal axis often shifts to the right and patients might experience problems such as postoperative dyspareunia or pelvic pain as a result of the shortening of the total vaginal length [23]. In the present study, no postoperative dyspareunia was encountered and sexual function improved postoperatively in both the vNOTES HUSLS and LLS groups. As these two techniques are both favorable in terms of preserving the vaginal length and axis, they are clearly distinguished from SSLF and they might be considered as appropriate techniques, especially for sexually active women. The OPTIMAL study revealed 5-year success rates of 64.5% and 63.1% for USLS and SSLF, respectively [24]. No recurrence of apical prolapse was observed during the follow-up period in the present study.

The improved surgical access and precise suture anchoring offered by vNOTES likely facilitated the superior postoperative Ba levels found in this study. This result was unexpected, as the mesh-augmented LLS procedure is typically associated with robust anterior support. The disparity may be explained by preoperative anatomical variations; the LLS group started with more advanced anterior descent (lower Ba points) and higher apical positions (higher C points). While these baseline differences were not statistically significant (p > 0.05), they may have limited the comparative degree of postoperative elevation in the LLS group.

Regarding the individual anatomical compartments of the vaginal wall (anterior, posterior, or apical), recurrent prolapses most often occur from the anterior vaginal wall, particularly in women with advanced prolapse. In the present study, anterior recurrence was observed in a total of 4 patients, with 2 in each group (6.25%). Since there was no significant difference in the incidence of anterior recurrence between the surgical intervention groups, the study results are in line with the results of other studies in the literature, including the OPTIMAL study [24].

According to the De Lancey classification, uterosacral and cardinal ligaments support the apex, cervix, and proximal vagina. Therefore, treatment of vault or uterine prolapse might also help cure cystocele as the pubocervical fascia is suspended by a mesh. In this way, unnecessary cystocele treatment can be prevented [25]. A study aiming to evaluate the vaginal axis by magnetic resonance imaging concluded that the postoperative vaginal axis was optimal after LLS [26]. The results of the present study align with these findings. However, further prospective studies are needed. The risk of mesh erosion was found to be 3.8% with a mean follow-up of 82.3 months after LLS. Researchers have suggested that the risk of mesh erosion due to posterior mesh application and tobacco use might be reduced with the use of appropriate mesh material [27]. In the present study, mesh erosion was not observed, most likely due to the medium-term follow-up period and the use of appropriate mesh material. Vaginal approaches have been embraced in gynecological practice for many years. Thanks to recent technological advances, VNOTES may become a more preferred method compared to minimally invasive laparoscopic surgery. Factors contributing to its use include the absence of scarring, no incision pain, and optimal cosmetic results [28]. Likewise, a study on vNOTES for sentinel lymph node applications predicted that this method would continue to develop and grow in popularity [29]. No iatrogenic ureteral injury was observed in the vNOTES HUSLS group in this study. Visualization of pelvic structures via vNOTES may eliminate the slightly increased risk of ureteral complications associated with USLS. The clinical utility of vNOTES HUSLS is expected to expand, and this method may become the primary surgical approach for POP as proficiency with the technique increases. Larger-scale long-term prospective studies are still needed to determine the optimal method for the treatment of POP, which is more common with increasing life expectancy. The present study has yielded results compatible with the literature in terms of recurrence and postoperative outcomes. The retrospective nature of this study constitutes a limitation. However, to the best of our knowledge, this study is the first in the literature to compare the vNOTES HUSLS and LLS methods, which constitutes its main strength. The same surgical team performed all operations to avoid possible bias. The exclusion of patients for whom additional surgery was needed due to anterior or posterior prolapse or SUI prevented confusion in the evaluation of recurrences. The results of the study’s medium-term follow-up evaluations are encouraging in terms of apical prolapsus surgeries.

While this study provides valuable insights, it is also important to acknowledge its limitations. The study is limited by its retrospective design, which is inherently prone to selection and recall bias. As patients were offered both vNOTES HUSLS and LLS and the final decision was based on both surgeon and patient preference, the groups may not have been perfectly comparable. Factors such as specific anatomical challenges or patient-related comorbidities may have influenced the choice of procedure, potentially skewing the success and complication rates reported. In addition, the number of patients who were eligible for and willing to undergo each surgical procedure was limited, which contributed to the small sample size. The average follow-up period of 21 months was relatively short for assessing the long-term effectiveness and safety of the surgical procedures.

Overall, this study provides promising evidence that vNOTES HUSLS and LLS are both effective and safe treatments for vault prolapse with comparable anatomical and functional outcomes, postoperative complications, and recurrence rates between the groups. However, the limitations of the study should be considered while interpreting the results. Future research with larger sample sizes and longer follow-up periods are needed to confirm these findings and to assess the long-term effectiveness and safety of these procedures.

## Figures and Tables

**Figure:**
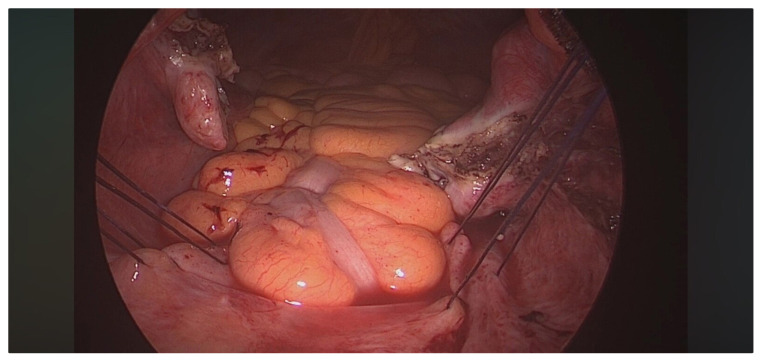
Bilateral nonabsorbable sutures were placed near the intermediate portions of the uterosacral ligament at the level of the ischial spine, constituting a total of 4 stitches.

**Table 1:** Baseline characteristics and preoperative POP-Q scores.

*Preoperative*	*vNOTES HUSLS*	*LLS*	
	Mean±SD	Mean±SD	p-value
***Age***	58.91±11.42	56.50±10.68	0.240
***BMI***	27.95±3.45	27.44±2.96	0.524
***Gravida***	4.39±2.72	4.97±3.69	0.480
***Parity***	3.52±2.57	4.28±3.63	0.339
***VD***	3.48±2.61	4.25±3.65	0.343
***CS***	0.35±0.76	0.34±0.75	0.953
***Perineal body***	2.99±0.15	3.32±0.31	0.067
***Genital hiatus***	4.02±0.47	3.95±0.36	0.539
***Aa***	0.66±2.04	0.63±2.42	0.352
***Ba***	1.06±3.58	2.06±3.61	0.270
***C***	1.72±2.98	0.72±3.07	0.191
***Ap***	−0.38±1.54	−1.53±2.13	0.051
***Bp***	0.75±2.20	−0.31±2.28	0.062
***TVL***	8.13±1.76	8.06±1.34	0.873

POP-Q: Pelvic Organ Prolapse Quantification; vNOTES HUSLS: vaginal natural orifice transluminal endoscopic surgery high uterosacral ligament suspension; LLS: laparoscopic lateral suspension; SD: standard deviation; BMI: body mass index; VD: vaginal delivery; CS: cesarean section; TVL: total vaginal length.

**Table 2:** POP-Q staging of patients preoperatively and at the 24th postoperative month and comparison of mean preoperative and postoperative PISQ-12 and FSFI scores between the groups.

	*vNOTES HUSLS*			*LLS*		
	Preoperative	Postoperative	p	Preoperative	Postoperative	p-value
***Aa***	−0.71±2.05	−2.03±0.94	<0.001	0.63±2.42	−1.97±0.82	<0.001
***Ba***	1.00±3.63	−1.97±1.05	<0.001	2.06±3.61	−1.03±2.10	0.017
***C***	1.72±2.98	−3.66±1.13	<0.001	0.72±3.07	−4.06±1.74	0.001
***Ap***	−0.38±1.54	−1.78±0.79	<0.001	−1.53±2.13	−2.19±0.97	0.001
***Bp***	0.75±2.20	−1.19±1.12	<0.001	−0.31±2.28	−1.78±0.66	<0.001
**Perineal body**	2.99±0.15	2.25±0.18	0.016	3.32±0.31	2.79±0.45	0.03
***Genital hiatus***	4.01±0.47	3.32±0.52	0.031	3.96±0.35	3.21±0.38	0.02
***TVL***	8.13±1.76	8.28±1.63	0.557	8.06±1.34	8.03±1.45	0.891
***PISQ-12***	18.88±2.59	36.16±2.67	<0.001	18.81±2.53	34.53±2.26	<0.001
***FSFI-desire***	3.13±0.68	5.02±0.52	<0.001	2.17±0.56	4.82±0.56	<0.001
***FSFI-arousal***	3.88±0.55	5.66±0.49	<0.001	3.29±0.26	5.87±0.50	<0.001
***FSFI-lubrication***	4.63±0.27	6.26±0.33	<0.001	4.69±0.22	6.55±0.33	<0.001
***FSFI-orgasm***	4.12±0.36	4.70±0.35	<0.001	4.18±0.87	4.50±0.26	<0.001
***FSFI-satisfaction***	3.98±0.55	5.71±0.30	<0.001	3.29±0.30	5.88±0.35	<0.001
***FSFI-pain***	5.32±0.21	6.26±0.69	<0.001	5.17±0.28	6.46±0.29	<0.001
***FSFI-total***	28.21±1.27	30.44±0.73	<0.001	26.26±0.63	30.63±1.02	<0.001

Variables are given as mean ± standard deviation. POP-Q: Pelvic Organ Prolapse Quantification; vNOTES HUSLS: vaginal natural orifice transluminal endoscopic surgery high uterosacral ligament suspension; LLS: laparoscopic lateral suspension; TVL: total vaginal length; PISQ-12: Pelvic Organ Prolapse/Urinary Incontinence Sexual Questionnaire; FSFI: Female Sexual Function Index.

**Table 3:** Perioperative outcomes and PGI-I scores.

Variables	vNOTES HUSLS (n=32)	LLS (n=32)	p-value
**Duration of Surgery, min (mean±SD, min-max)** *	85.48±25.65 (50–120)	91.16±32.49 (60–130)	0.826
**Drop in hemoglobin levels, g/dL (mean±SD, min-max)**	1.1±0.7 (0.5–2.3)	1.1±0.8 (0.5–2.2)	0.891
**Postoperative complications, n (%) hip pain**	0 (0)	1 (3.1)	0.997
**Length of hospital stay (day) (median (IQR), min-max )**	1.5 (1) (1–2)	2 (1) (1–3)	0.122
**PGI-I score**	1.6±0.8	1.7±0.7	0.344

Variables are presented as mean±standard deviation or median (interquartile range) according to distribution characteristics. PGI-I: Patient Global Impression of Improvement; vNOTES HUSLS: vaginal natural orifice transluminal endoscopic surgery high uterosacral ligament suspension; LLS: laparoscopic lateral suspension; SD: standard deviation; IQR: interquartile range.

* Duration of surgery, median (IQR): vNOTES HUSLS, 85 (60–105) min; LLS, 90 (65–120) min.

**Table 4:** Postoperative comparison of vNOTES and LLS.

*Postoperative*	*vNOTES HUSLS*	*LLS*	
	Mean±SD	Mean±SD	p-value
***PISQ-12***	36.16±2.67	34.53±2.26	0.501
***FSFI-desire***	5.02±0.52	4.82±0.56	0.133
***FSFI-arousal***	5.66±0.49	5.87±0.50	0.055
***FSFI-lubrication***	6.26±0.33	6.55±0.33	0.298
***FSFI-orgasm***	4.70±0.35	4.50±0.26	0.444
***FSFI-satisfaction***	5.71±0.30	5.88±0.35	0.239
***FSFI-pain***	6.26±0.69	6.46±0.29	0.061
***FSFI-total***	30.44±0.73	30.63±1.02	0.396
***Perineal body***	2.25±0.18	2.79±0.45	0.001
***Genital hiatus***	3.32±0.52	3.21±0.38	0.539
***Aa***	−2.03±0.94	−1.97±0.82	0.777
***Ba***	−1.97±1.05	−1.03±2.10	0.030
***C***	−3.66±1.13	−4.06±1.74	0.272
***Ap***	−1.78±0.79	−2.19±0.97	0.071
***Bp***	−1.19±1.12	−1.78±0.66	0.052
***TVL***	8.28±1.63	8.03±1.45	0.123

vNOTES HUSLS: Vaginal natural orifice transluminal endoscopic surgery high uterosacral ligament suspension; LLS: laparoscopic lateral suspension; PISQ-12: Pelvic Organ Prolapse/Urinary Incontinence Sexual Questionnaire; FSFI: Female Sexual Function Index; TVL: total vaginal length.
